# Who fails to return within 30 days after being tested positive for HIV/STI in a free testing centre?

**DOI:** 10.1186/s12879-020-05520-7

**Published:** 2020-10-27

**Authors:** Camille Rolland, Elise de La Rochebrochard, Prescillia Piron, Marc Shelly, Christophe Segouin, Pénélope Troude

**Affiliations:** 1 Department of Public Health, University Hospital Saint-Louis-Lariboisière-Fernand-Widal, AP-HP, 200 rue du Faubourg-Saint-Denis, F-75475, Paris Cedex 10, Paris, France; 2 Free Sexual Health Centre, University Hospital Saint-Louis-Lariboisière-Fernand-Widal, AP-HP, Paris, France; 3 Institut National d’Etudes Démographiques (Ined), Aubervilliers, France; 4 University Paris-Saclay, University Paris-Sud, UVSQ, CESP, INSERM, Paris, Villejuif France

**Keywords:** HIV infection, STI, Screening, Preventive health, Sexual risk behaviours, Patient dropouts

## Abstract

**Background:**

Some patients who test positive for sexually transmitted infections (STIs) fail to return for results and treatment. To target improvement actions, we need to find out who these patients are. This study aimed to explore factors associated with failure to return within 30 days (FTR30) after testing among patients with positive results in a free STI testing centre in Paris.

**Methods:**

All patients with at least one positive result between October 2016 and May 2017 and who completed a self-administered questionnaire were included in this cross-sectional study (*n* = 214). The questionnaire included sociodemographic factors, sexual behaviour and history of testing. Factors associated with FTR30 were assessed using logistic regression models.

**Results:**

More than two-thirds of patients were men (72%), and the median age of patients was 27 years. Most patients were born in metropolitan France (56%) or in sub-Saharan Africa (22%). Men who had sex with men represented 36% of the study population. The FTR30 rate was 14% (95% CI [10–19%]). In multivariate analysis, previous HIV testing in younger persons (aOR: 3.36, 95% CI [1.27–8.84]), being accompanied by another person at the pretest consultation (aOR: 3.45, 95% CI [1.36–8.91]), and lower self-perceived risk of HIV infection (aOR: 2.79, 95% CI [1.07–7.30]) were associated with a higher FTR30. Testing for chlamydia/gonorrhoea without presumptive treatment was associated with a lower FTR30 (aOR: 0.21, 95% CI [0.07–0.59]).

**Conclusions:**

These factors that affect failure to return are related to the patient’s representations and involvement in the STI screening process. Increasing health literacy and patient empowerment could help to decrease failure to return after being tested positive for HIV/STI.

**Trial registration:**

Not applicable.

## Introduction

Sexually transmitted infections (STIs) are a major challenge in public health as nearly one million people become infected every day in the world with chlamydia, gonorrhoea, syphilis or trichomoniasis [1]. To decrease the spread of STIs, the World Health Organization (WHO) recommends focusing particularly on screening, counselling and early treatment [2]. In France, these missions are carried out by the national network of free testing centres for sexually transmitted infections. However, a proportion of patients who are tested for STIs do not return for their results. Some studies have suggested that the fear of a positive HIV result, stigmatization and discrimination may explain failure to return for test results [3–5]. For patients screened positive, failure to return (FTR) means that they are not aware of their positive result. Consequently, they cannot begin early treatment and receive additional information.

Some studies that explored FTR after HIV/STI tests reported rates that ranged from 3 to 30% [6–11]. Results concerning factors associated with FTR appeared mixed but suggested that FTR is affected by both individual factors and contextual factors (access to testing, social stigma …) [12]. Most previous studies on FTR were conducted among the total patient population whatever the result of screening. However, some studies suggested that patients with a positive test are less likely to return than patients with negative results [12]. Moreover, none of the previous studies took into account full STI screening (HIV, HBV, HCV, syphilis, chlamydia and gonorrhoea). The recent introduction of pre-exposure prophylaxis (PrEP) for HIV raises concerns about STI incidence and the possible decline in condom use [13]. Besides, use of rapid point-of-care testing (POCT) for HIV may increase the FTR for other tested STIs [9]. In view of these new prevention strategies, FTR is now a concern for all STIs and not only for HIV.

In order to decrease FTR for patients who screen positive, a first approach would be to improve understanding of patient-related factors that influence FTR and to develop improvement actions for these high-risk patients [8, 12, 14]. Another approach consists in developing new ways of contacting patients with their results, including SMS notification [10, 11, 15]. The first international trials showed that SMS notification is associated with a shorter time before starting treatment [10, 11]. Since August 2016, SMS notification is being tested in a free STI testing centre in Paris [16]. If at least one result is positive, the SMS invites the patient to return to the centre to obtain their results and receive appropriate guidance and/or treatment. This procedure is in agreement with national and European guidelines as positive results are still always delivered in person by a physician [17].

The objective of this study was to explore factors associated with failure to return within 30 days after testing among persons with at least one positive result in a free STI testing centre in Paris, France, which offers SMS notification.

## Materials and methods

### Setting

The study was conducted in a free STI testing centre in Fernand Widal Hospital in northern Paris. Care is entirely free of charge for all patients, even if they have no health insurance coverage. The tests performed include HIV, HBV, HCV, syphilis, gonorrhoea and chlamydia (urinary, vaginal, oropharyngeal and anorectal tests). HIV, hepatitis and syphilis are screened by serology. For gonorrhoea and chlamydia screening, self-swabbing is done by patients. Before the consultation, a self-administered questionnaire was proposed to all new patients visiting the centre and was completed in the waiting room. This questionnaire was included in the patient’s medical file and helped the doctor to direct the consultation according to the patient’s answers. However, only patients familiar with written French were able to complete the questionnaire. During pre-test counselling, the doctor evaluates each patient’s risk-taking behaviour and prescribes appropriate screening tests. Moreover, according to national recommendations for chlamydia/gonorrhoea, treatment is systematically started prior to results (presumptive treatment) when symptoms are strongly suggestive of chlamydia/gonorrhoea infections. Results are available approximately 1 week later. Patients can choose to be tested anonymously or not. Whether the patient chooses to remain anonymous for screening or not, he or she is given an anonymity number ensuring confidentiality throughout the process. The text messaging programme is proposed to patients by the reception agent who explains the programme with the help of a leaflet: 1) if all results are negative, the patient is informed by SMS and he/she does not need to return to the testing centre; 2) if at least one result is positive, the SMS invites the patient to come back to the centre to obtain the results. Up to three messages are sent if the patient fails to return. The SMS programme has been developed to respect medical confidentiality and is presented in detail elsewhere [16].

### Study population

From October 2016 to May 2017, 2611 patients visited the free STI testing centre. During the 8 months of the study period, 2315 patients underwent STI testing, 219 came for post-exposure treatment and 77 came only for information with no testing. Among the 2315 patients who underwent STI testing, 266 (11%) patients had at least one positive test. Of these 266 positive patients, 214 patients filled in the self-administered questionnaire (participation rate 80%) and were included in this cross-sectional study.

### Data sources

Data were collected from two complementary sources: the consultation database and the self-administered questionnaire.

The following data were routinely entered in the consultation database: date of first visit, anonymous testing, participation in the text messaging programme, STIs tested, provision of presumptive treatment for chlamydia or gonorrhoea or not, date of return, and results of the screening tests performed.

The questionnaire included sociodemographic factors: age, gender (woman, man, transgender), place of residence, place of birth, educational level and health insurance coverage, sexual behaviour and history (including sexual orientation and occasional and/or regular sex partners), previous HIV testing and factors related to the visit to the centre (who suggested testing, accompaniment by another person at the consultation, reason for attending indicated by one or several items that can be ticked among a list of 14 reasons), self-perceived risk of HIV infection (the patient perceived himself/herself at a higher risk of infection compared with the general population, equal risk, lower or no risk).

### Outcome

In the literature, the time period used to define FTR varied between 1 month and 1 year [4, 9, 12, 14]. However, all studies showed that most patients returned for results between 7 days and 21 days after testing [6, 8, 10, 18, 19]. Thus, FTR at 30 days is a relevant indicator and was used in this study.

### Factors analysed

Only 10 patients were aged over 25 years and had no previous HIV testing. Age and previous HIV testing were therefore combined in one variable with three categories: *patients aged 25 years or older, patients under 25 years with no previous HIV testing and patients under 25 years with previous HIV testing*.

In our study population, there were no transgender persons and only 7 patients were women who had sex with women. Gender and sexual orientation were therefore combined in a single variable with three categories: *men who had sex with men (including bisexual men), heterosexual men, and women*.

To explore both the potential role of self-swabbing and of presumptive treatment in FTR (patients may have thought they did not need to return because they had already been treated), data on testing for chlamydia/gonorrhoea infections and provision of presumptive treatment at the pre-test visit were combined in a single variable: *not tested for chlamydia and gonorrhoea, tested without provision of presumptive treatment, tested with provision of presumptive treatment*.

Consultation for risk-taking was based on the reason for attending given by patients in the questionnaire. We considered that the consultation was for risk-taking if the patient ticked at least one item among the 11 reasons corresponding to a risk (unprotected sexual intercourse, clinical signs of STI, HIV-positive sex partner, sex with sex worker …). The consultation was not for risk-taking if the patient reported that he/she wanted only information or reassurance, or if the patient wanted to be tested before stopping condom use with his/her regular partner.

Self-perceived risk of HIV infection was summarised into a binary variable: *self-perceived risk higher or equal to the general population* vs *lower or no risk*.

### Statistical methods

Patient characteristics were compared according to FTR30 using the *χ*^**2**^ test or Fisher’s test. Univariate and multivariate analyses were conducted to assess factors associated with FTR30 using logistic regression models. Considering the limited number of events in our study, we adopted a parsimonious methodology using a stepwise backward selection with a threshold of 0.2 for removal from the model. Association was considered as statistically significant when *p* < 0.05. Statistical analyses were performed using STATA/SE 11.0 (Stata Corporation, College Station, TX, USA).

## Results

The characteristics of the study population are presented in Table 1. The median age of patients was 27 years and more than two-thirds were men (72%). Men who had sex with men (MSM) represented 36% of the study population. More than half of the participants lived in Paris. Most patients were born in metropolitan France (56%) or in sub-Saharan Africa (22%), and more than three-quarters of the population had completed education beyond high school (79%). Most patients had already been tested for HIV (84%), less than half declared that they had occasional sex partners (43%) but two-thirds declared that they came for screening because of risk-taking (67%). The majority of patients preferred to remain anonymous (64%) but agreed to be notified by SMS after screening (72%).

**Table 1:** Description of the study population (*n* = 214)

Characteristics	n	%
Age* (years) and previous HIV testing		
≥ 25	132	62
< 25 and no previous HIV testing	25	12
< 25 and previous HIV testing	57	26
**Sexual orientation**		
Men who had sex with men (MSM)	76	36
Heterosexual men	77	36
Women	61	28
**Place of residence**		
Paris	132	62
Outside Paris	82	38
**Birthplace**		
Metropolitan France	120	56
Sub-Saharan Africa	47	22
Overseas France or abroad	47	22
**Level of education**		
High school graduate	170	79
Not a high school graduate	44	21
**Health insurance coverage**		
Statutory health insurance	172	80
State assistance	23	11
None	19	9
**Occasional partners**		
No	123	57
Yes	91	43
**Self-perceived risk of HIV infection**		
Equal or greater than other people	100	47
Less than other people or no risk	114	53
**Consultation for risk-taking**		
No	71	33
Yes	143	67
**Accompanied by another person**		
No	169	79
Yes	45	21
**Anonymous testing**		
Yes	136	64
No	78	36
**Participation in SMS programme**		
Yes	155	72
No	59	28
**Chlamydia/gonorrhoea testing**		
Not tested for chlamydia/gonorrhoea	35	16
Chlamydia/gonorrhoea testing with presumptive treatment	19	9
Chlamydia/gonorrhoea testing without presumptive treatment	160	75

*Category ≥25 includes patients aged 25 to 70 years; category < 25 includes patients aged 18 to 24 years

Patients were screened for a median of 5 STIs among the 6 available in the centre (Q1-Q3 [4–6]). Most patients were positive for only one STI (91%), 18 patients were positive for two (8%) and one patient for three. Distribution of positive screenings is presented in Table 2. Of the 214 patients tested positive for at least one STI and included in the study, 14% (*n* = 30) failed to return within 30 days after testing (95% CI [10–19%]). All patients who tested positive for HIV (*n* = 10) returned within 30 days after testing. However, over a quarter of patients who tested positive for HBV failed to return for their results (8/29). Of the 30 patients who failed to return within 30 days, 7 returned between 31 and 60 days. The 60-day FTR rate was 11% (95% CI [7–16%]). Overall, the median interval before returning for results was 8 days (Q1–Q3 [6–13]).

**Table 2:** Distribution of positive screenings and failure to return for results (*n* = 214)

Positive screenings	n	%	FTR30/n
Chlamydia	107	50	13/107
Gonorrhoea	31	14	3/31
HBV	26	12	7/26
Chlamydia and gonorrhoea	14	7	2/14
HCV	12	6	1/12
Syphilis	11	5	3/11
HIV	8	4	0/8
Chlamydia and HBV	2	1	0/2
HIV and syphilis	1	0.5	0/1
HIV and syphilis and HCV	1	0.5	0/1
Gonorrhoea and HBV	1	0.5	1/1
Total	214	100	30/214

Table 3 presents factors associated with FTR30. In univariate analysis, FTR30 appeared higher for patients younger than 25 years old who had previous HIV testing (23%) compared with patients under 25 years old with no previous HIV testing (4%). The FTR30 rate was higher for patients who had a lower self-perceived risk of HIV infection (20%) than for those who saw themselves as being at higher risk (7%, chi2 *p* = 0.008). The FTR30 rate was 24% for patients who were accompanied versus 11% for patients who came to the centre alone (chi2 *p* = 0.03). Patients who were tested for chlamydia/gonorrhoea and were given presumptive treatment were twice as likely to fail to return for their results as patients who were tested without provision of presumptive treatment (21% vs. 10%). The FTR30 rate reached 29% for patients not tested for chlamydia/gonorrhoea. Of the 155 patients who agreed to be notified by SMS, 12% failed to return whereas this percentage was 20% among the 59 patients who did not agree, but this difference was not statistically significant (chi2 *p* = 0.10). The FTR30 rate was not significantly associated with gender, birthplace, educational level, sexual orientation, occasional partners, or testing linked to risk-taking.

**Table 3:** Factors associated with FTR for HIV/STI test results

Factors	FTR30/n	FTR (%)	Univariate analysis			Multivariate analysis*		
			OR	95% CI	***P*** value	OR	95% CI	***P*** value
**Age (years) and previous HIV testing**					0.06			0.01
≥ 25	16/132	12	1			1		
< 25 and no previous HIV testing	1/25	4	0.30	0.04–2.39		0.31	0.03–2.87	
< 25 and previous HIV testing	13/57	23	2.14	0.95–4.81		3.36	1.27–8.84	
**Birthplace**					0.80			–
Metropolitan France	16/120	13	1			–		
Sub-Saharan Africa	8/47	17	1.33	0.53–3.36		–	–	
Overseas France or abroad	6/47	13	0.95	0.35–2.60		–	–	
**Level of education**					0.37			–
High school graduate	22/170	13	1			–		
Not a high school graduate	8/44	18	1.49	0.62–3.63		–	–	
**Health insurance coverage**					0.52			–
Statutory health insurance	24/172	14	1			–	–	
State assistance	2/23	9	0.59	0.13–2.67		–	–	
None	4/19	21	1.64	0.50–5.37		–	–	
**Sexual orientation**					0.08			–
MSM	5/76	7	1			–		
Heterosexual men	14/77	18	3.16	1.08–0.25		–	–	
Women	11/61	18	3.12	1.02–9.55		–	–	
**Occasional partners**					0.76			–
No	18/123	15	1			–		
Yes	12/91	13	0.89	0.40–1.95		–	–	
Self-perceived risk of HIV infection**					< 0.01			0.04
Equal or greater than other people	7/100	7	1			1		
Less than other people or no risk	23/114	20	3.35	1.37–8.21		2.79	1.07–7.30	
**Consultation for risk-taking**					0.21			–
No	13/71	18	1			–		
Yes	17/143	12	0.60	0.27–1.32		–	–	
**Accompanied by another person**					0.03			0.01
No	19/169	11	1			1		
Yes	11/45	24	2.55	1.11–5.86		3.45	1.36–8.91	
**Anonymous testing**					0.10			–
Yes	15/136	11	1			–		
No	15/78	19	1.92	0.88–4.18		–	–	
**Participation in SMS programme**					0.10			–
Yes	18/155	12	1			–		
No	12/59	20	1.94	0.87–4.33		–	–	
**Chlamydia/gonorrhoea testing**					0.02			< 0.01
Not tested for chlamydia/gonorrhoea	10/35	29	1			1		
Chlamydia/gonorrhoea testing with presumptive treatment	4/19	21	0.67	0.18–2.51		0.55	0.12–2.56	
Chlamydia/gonorrhoea testing without presumptive treatment	16/160	10	0.28	0.11–0.68		0.21	0.07–0.59	

*Variables selected by a stepwise backward selection with a threshold of 0.2 for removal from the model

**Compared with the general population

Among all factors studied in univariate analyses, the stepwise backward selection retained four factors: age combined with previous HIV testing, self-perceived risk of HIV infection, being accompanied and chlamydia/gonorrhea testing. In multivariate analysis, patients aged 25 or under who had previously had HIV testing were more likely to fail to return than patients over 25 years old (aOR: 3.36, 95% CI [1.27–8.84]). A lower self-perceived risk of HIV infection remained significantly associated with a higher FTR30 in multivariate analysis (aOR: 2.79, 95% CI [1.07–7.30]), as well as being accompanied (aOR: 3.45, 95% CI [1.36–8.91]) compared with patients coming alone. Being tested for chlamydia/gonorrhoea without treatment was associated with a lower FTR30 compared with patients not tested for chlamydia/gonorrhoea (aOR: 0.21, 95% CI [0.07–0.59]).

Fifty-two patients were not included in this study as they had not completed the self-administered questionnaire. These patients tended to be older than the study population (proportion of patients ≥25 years was 81% vs 62%, chi2 *p* = 0.01). The proportion of women did not differ between included and excluded patients (29% vs 17%, chi2 *p* = 0.10). FTR30 was 17% among excluded patients and was not significantly different from that observed in the study population (14%, chi2 *p* = 0.54).

## Discussion

In our centre from October 2016 to May 2017, 14% of the 214 patients who screened positive for at least one STI and were included in the study failed to return within 30 days. As expected, the FTR within 30 days did not differ from the FTR within 60 days (14 and 11%, respectively), as the majority of patients returned between 6 and 13 days. One possible limitation of our study is the exclusion of 52 patients out of 266 tested positive (20%) because they did not complete the self-administered questionnaire. These excluded patients could have less favourable social characteristics (more often non-French speaking patients, less educated patients not at ease with a written questionnaire). However, examination of the consultation database showed that among patients who were screened positive, the FTR30 rate did not differ significantly between those who were included in the study and those who were not included. The FTR rate differed between HIV (0 FTR) and other STIs (from 1/12 to 7/26). As most previous studies on FTR included all patients whatever the result of screening and generally studied only one STI, comparison with our findings must be cautious. Regarding HIV testing, previous studies in similar STI centres reported FTR rates that ranged from 7 to 22% [9, 12, 14]. The low FTR that we observed among HIV-positive patients is therefore very encouraging. Regarding testing for other STIs, previous studies reported FTR rates that ranged from 5 to 29% according to the STI tested (chlamydia, hepatitis or syphilis) and the setting [9, 20]. More specifically, FTR rates after chlamydia testing ranged between 17 and 29% [9, 20]. Despite the limitations we have previously noted, with the exception of HIV our findings thus seem consistent with FTR rates reported in the literature. These findings must be confirmed in larger studies. Nevertheless, they demonstrate the need to take into account all STIs and not only HIV when considering FTR, especially for positive patients.

One approach to decreasing the FTR is to diversify the options for contacting the patient for their results [10, 11, 15]. The option developed in our centre was to propose SMS notification after screening [16]. Although the acceptability of this programme appeared good, this study was not able to demonstrate a statistically significant difference in FTR30 between participants (tested positive for at least one STI) and non-participants in the SMS programme. The impact of SMS notification on failure to return needs to be assessed in a larger sample and diversification of contact options must be pursued to meet the preferences of patients [21–23]. Participation in programmes such as SMS notification may differ according to patients’ profiles, such as age and social characteristics [16]. Factors related to the screening structure or screening modalities may affect the decision to undertake STI screening [24, 25]. For instance, patients could also be offered systematic phone calls after testing to reduce the risk of failure in delivering STI screening results [9].

A second approach to decreasing the FTR is to target improvement actions for patients who are at high risk of failure to return for results. In this study, none of the sociodemographic factors analysed was significantly associated with FTR30. However, persons younger than 25 years old who had previous HIV testing were less likely to return for results than others. This was an unexpected finding and several hypotheses could be put forward. One reason could be fear about the results linked to a bad experience during previous testing. Conversely, it is possible that younger persons may think that they do not need to worry about the results because someone will try to contact them if necessary. The higher risk of FTR30 among younger patients with previous HIV testing might also be explained by erroneous beliefs or misconceptions regarding STI risk and prevention, as if STI screening was a protective factor even without knowing the result, especially if previous testing was negative. This hypothesis is supported by the results of a French study evaluating trends in HIV-related knowledge, risk perceptions and sexual behaviours that showed an increase in erroneous beliefs regarding HIV transmission between 1994 and 2010 among young adults (18–29 years old) [26]. Moreover, young adults were less convinced of the efficacy of condoms in protecting against HIV (70–80% in 1992–1994 vs. 50% in 2010) and the proportion of respondents who were very afraid of AIDS significantly decreased (from 44 to 20%). The decrease in the level of knowledge regarding HIV transmission and prevention over the last decades explains the decrease in adopting prevention practices by young people (condom use, contraception use...) [26]. This demonstrates the need to heighten awareness amongst patients, especially younger ones, during the pre-test counselling on STI prevention and the need to insist that it is imperative to return for results [26].

In this study conducted in 214 patients screened positive for at least one STI, more than half of the patients (53%) felt that they had a lower risk or no risk of HIV infection compared with the general population. This result highlights the discrepancy between real risk taken and perceived risk. Patients with a lower self-perceived risk of HIV infection were more likely to fail to return for their results. This finding is in agreement with previous studies that reported an association between FTR and a lower perceived risk for HIV [12]. The absence of awareness of risk taken among patients screened positive for STIs is a clear challenge in STIs and HIV prevention, as it affects the probability that these patients will return for their results and thus their chance of being quickly treated and avoiding new infections. It would be of primary importance to investigate this discrepancy and especially the impact of health literacy. This perceived low risk of HIV infection could in fact reflect a lack of knowledge or more precisely a low health literacy regarding STIs [27]. Health literacy represents the degree to which individuals have an understanding of health information in order to make appropriate health decisions [28, 29]. Patients with low literacy are less likely to understand prevention, treatment and follow-up of STIs and are often at high risk of infection [28, 30]. Health literacy has also been associated with willingness to comply with healthcare providers’ recommendations relative to STIs [27, 29]. Lastly, difficulties in understanding and acting on health information about STIs may negatively influence disease prevention and in particular may increase the likelihood of failure to return for post-test counselling [29]. Moreover, studying the difference in the second category, by differentiating “higher risk” and “equal risk”, could provide additional information.

Testing for chlamydia and gonorrhoea without presumptive treatment at the first visit was associated with a lower risk of FTR30 compared with patients not tested for chlamydia/gonorrhoea. Unlike other infections that are screened on a blood sample taken by a nurse, testing for chlamydia and gonorrhoea is based on self-swabbing, making the patient a direct actor in their own screening. Self-swabbing could be an important factor of patient empowerment, as has been suggested in cervical cancer screening [31] and streptococcus testing [32]. In line with these results, the lower FTR30 observed in our study among self-swabbing patients tested for chlamydia and gonorrhoea could reflect a greater empowerment of these patients. This lower risk of FTR30 was not observed for patients presumptively treated. This result was expected as patients believe they are already cured.

The FTR30 in our study was also higher among patients who were accompanied by another person compared with patients who came alone. No data were found in the literature on this factor. A possible hypothesis is that patients who had been motivated to come for screening by their peers may lose the benefit of the group effect when coming back for results, and so are less likely to return.

## Conclusion

In conclusion, factors that affect failure to return (previous HIV testing, self-perceived risk of HIV infection, testing for chlamydia/gonorrhoea and being accompanied) are related to the representations and involvement of the patients in the HIV/STI screening process. They are strongly related to patients’ health literacy that directly influences health knowledge, health status, risk-taking behaviours and use of health services [29, 30].

To decrease failure to return after HIV/STI screening, different approaches could be considered: one approach consists in adapting the screening offer proposed by the centre, another is centred on patients defined as having a high-risk profile. In our study, we tested the impact of SMS notification but this is only one first step to facilitate contact with patients and it is necessary to pursue the diversification of contact options. A profile of patients at high risk of non-return was highlighted: all factors associated with FTR30 (previous HIV testing among younger patients, being accompanied, no testing for chlamydia/gonorrhoea, lower self-perceived risk of HIV infection) were related to the representations and involvement of the patient in the STI screening process. Increasing the patients’ level of knowledge of STIs and their level of health literacy may be a way to reduce the risk of failure to return for test results and thus to empower patients [28].

## Data Availability

The datasets analysed during the current study are not publicly available due to patient confidentiality but are available from the corresponding author after having fulfilled French legal mandatory declarations to access hospital private data.

## Abbreviations

AIDS: Acquired immune deficiency syndrome
aOR: Adjusted odds ratio
CI: Confidence interval
HBV: Hepatitis B virus
HCV: Hepatitis C virus
HIV: Human immunodeficiency virus
FTR: Failure to return
FTR30: Failure to return within 30 days after testing
MSM: Men who have sex with men
OR: Odds ratio
PrEP: Pre-exposure prophylaxis
POCT: Point-of-care testing
SMS: Short message service
STIs: Sexually transmitted infections
WHO: World health organization
